# Early Stroke in a Young Male: Could it be Cerebral Autosomal Dominant Arteriopathy With Subcortical Infarcts and Leukoencephalopathy (CADASIL)?

**DOI:** 10.7759/cureus.103432

**Published:** 2026-02-11

**Authors:** Ajeet Raj, Sultan Ali, Muqaddas Imran, Karan Chaman Lal

**Affiliations:** 1 General Internal Medicine, Northampton General Hospital NHS Trust, Northampton, GBR; 2 Medicine, Liaquat University of Medical and Health Sciences, Jamshoro, PAK

**Keywords:** blood pressure, cerebral autosomal dominant arteriopathy with subcortical infarcts and leukoencephalopathy (cadasil), dizziness, facial droop, headache, hypertension, notch3 gene1, slurred speech, stroke, tongue-tie

## Abstract

A man in his early 40s presented with a sudden-onset headache, slurred speech, drooling, and a transient left-sided facial droop, which resolved within a few hours. Cerebral autosomal dominant arteriopathy with subcortical infarcts and leukoencephalopathy (CADASIL) is a rare monogenic hereditary cerebrovascular disorder that can cause stroke in adults due to mutations in the NOTCH3 gene and is associated with characteristic white matter changes on imaging (the “NOTCH” part of the gene name is derived from the *Drosophila melanogaster* notched wing mutant, and “3” signifies that it is the third discovered human homologue). The presence of elevated blood pressure raised questions regarding an alternative etiology for stroke. Therefore, he was referred to the accident and emergency department for further evaluation, including an MRI of the brain, which showed an acute infarct with periventricular white matter changes involving the right internal capsule. This report highlights the importance of considering CADASIL as an underlying cause of stroke in patients with persistent symptoms and characteristic white matter changes.

## Introduction

Stroke is a complex, multifactorial, and polygenic disease, and a serious life-threatening condition that leads to significant mortality and morbidity worldwide, with ischemic stroke being the most common type [1]. Many risk factors have been identified, such as hypertension, diabetes, and other pathological conditions. However, these factors are more commonly associated with elderly individuals, as among adults, 35-42% of strokes occur with an undefined cause. In rare cases, stroke in adults can also be caused by cerebral autosomal dominant arteriopathy with subcortical infarcts and leukoencephalopathy (CADASIL), a monogenic hereditary cerebrovascular disease caused by mutations in the NOTCH3 gene, and may present with characteristic white matter changes on imaging [2]. This report describes the case of a middle-aged man with a transient neurological event who was ultimately diagnosed with an ischemic stroke and suspected to have underlying CADASIL due to the presence of bilateral white matter lesions.

## Case presentation

**Figure 1:**
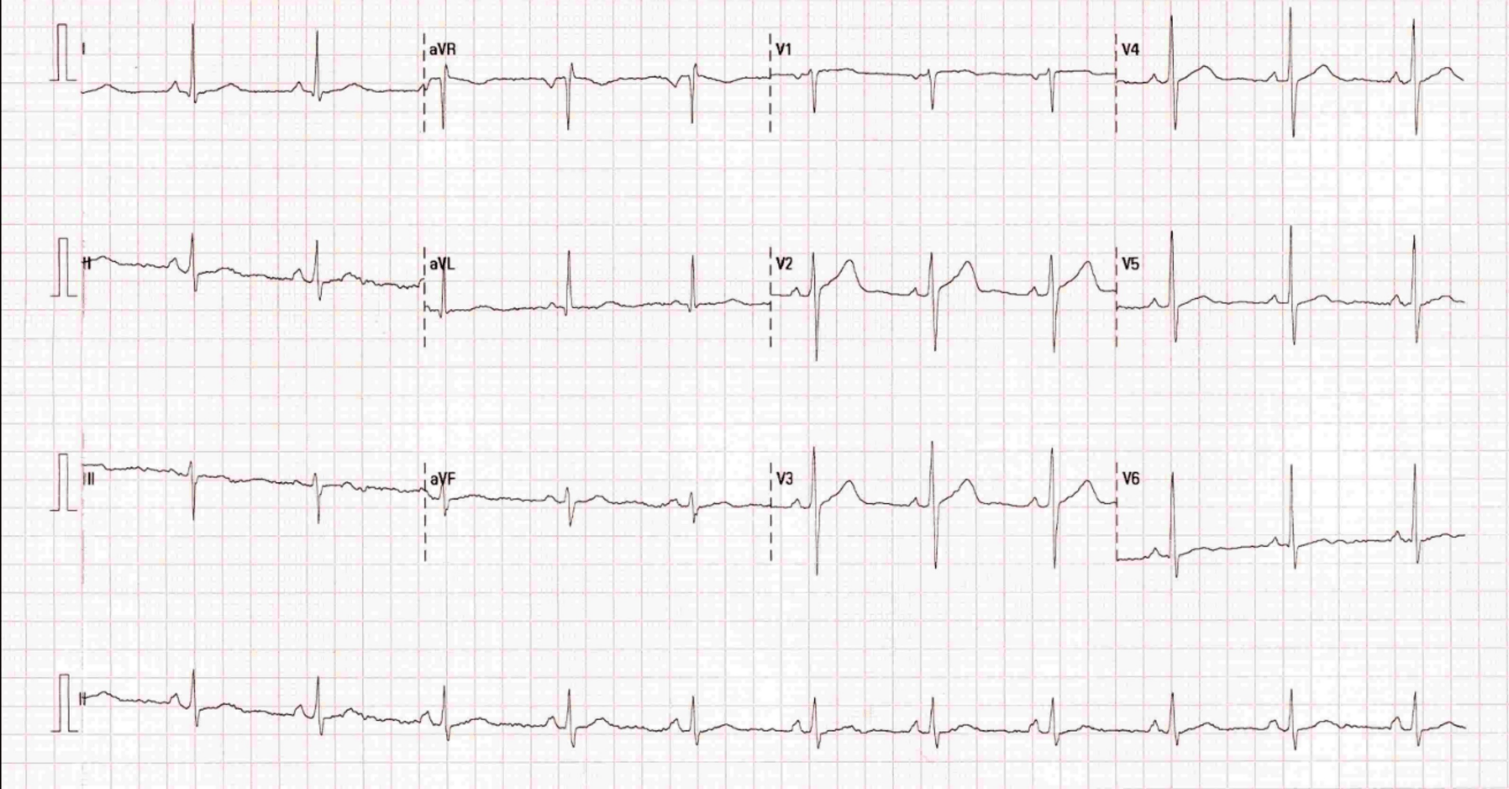
ECG showing a normal sinus rhythm and mild left ventricular hypertrophy ECG: electrocardiogram

A man in his early 40s with a history of diabetic retinopathy presented to his general practitioner (GP) with sudden-onset severe frontal and occipital headaches, slurring of speech, facial drooping, and drooling from the left corner of his mouth. His symptoms began at 10:00 am and resolved spontaneously by 2:00 pm, but he continued to experience a sensation of a tied tongue and a persistent headache. During the GP assessment, his blood pressure was 169/123 mmHg, and he was urgently referred to the Accident and Emergency (A&E) department for further evaluation of his hypertension. His observations in A&E showed a blood pressure of 176/129 mmHg, a heart rate of 86 beats per minute, and an oxygen saturation of 99% on room air. On examination, he was alert and oriented with a Glasgow Coma Scale (GCS) score of 15/15 and normal speech. However, he had right-sided weakness and a transient left-sided facial droop. The ECG showed mild left ventricular hypertrophy (Figure 1). His signs and symptoms were managed according to protocol. An urgent CT scan of the head was requested to rule out the cause of the raised blood pressure and a suspected underlying cerebrovascular event.

Investigations

**Figure 2:**
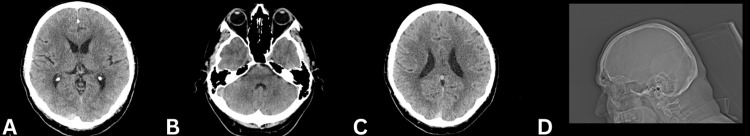
CT head scan (A, B, and C) Axial slices of the brain at different levels showing bilateral periventricular white matter hypodensities, more prominent in the right frontal lobe. (D) Sagittal (side view) reconstruction of the skull and brain CT: computed tomography

A CT scan showed no acute intracranial hemorrhage or recent infarction. However, bilateral periventricular white matter hypodensities were identified, more prominent in the right frontal lobe, on imaging (Figure 2).

**Figure 3:**
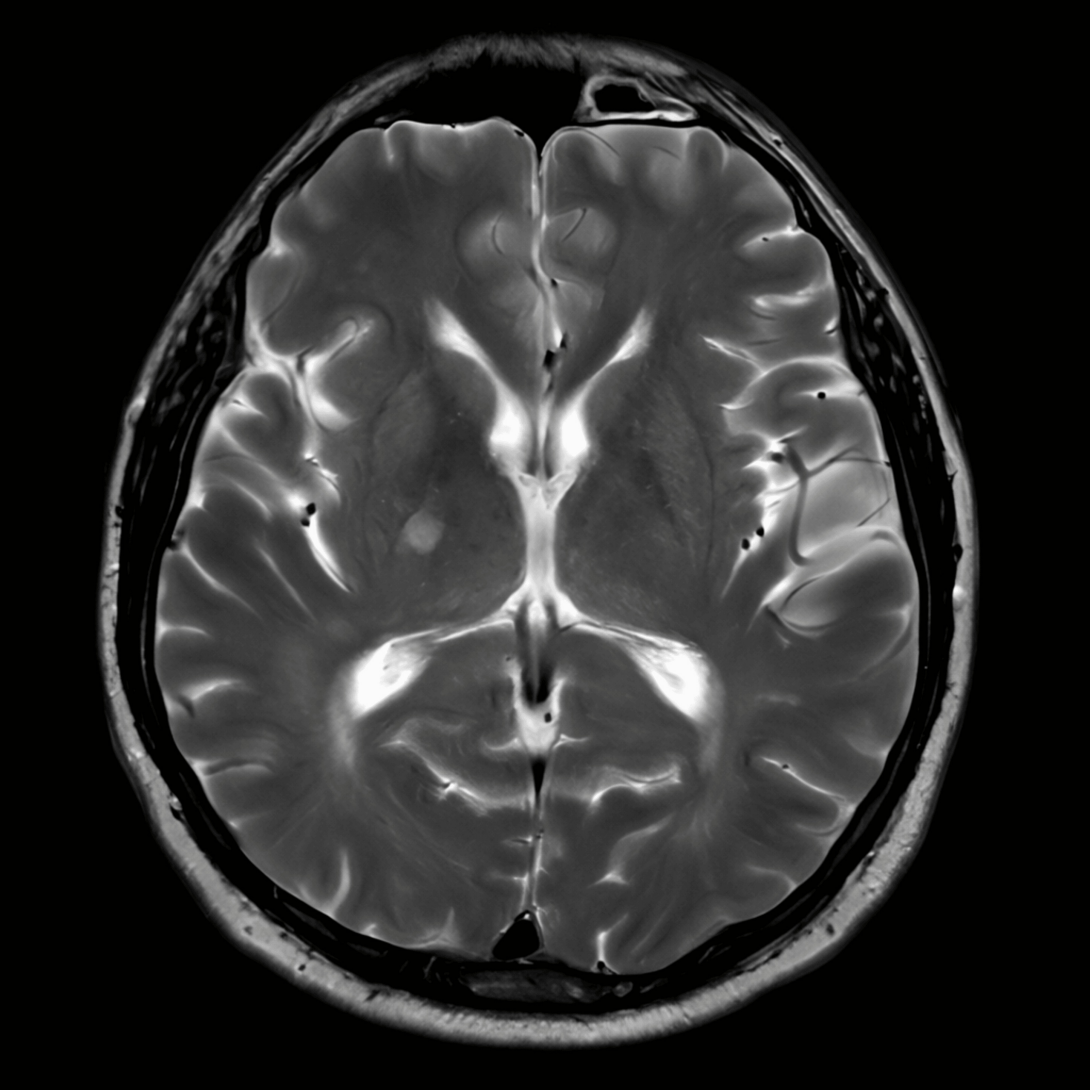
MRI head The image showed T2/FLAIR hyperintensities in the bilateral periventricular and deep white matter, with a small area of restricted diffusion adjacent to the posterior limb of the right internal capsule, consistent with a small area of ischemic infarction MRI: magnetic resonance imaging; FLAIR: fluid-attenuated inversion recovery

MRI head showed T2/FLAIR hyperintensities in the bilateral periventricular and deep white matter, with a small area of restricted diffusion adjacent to the posterior limb of the right internal capsule, consistent with a small area of ischemic infarction (Figure 3).

While the patient's blood tests were unremarkable, including the full blood count, the renal function, and the lipid profile, his cholesterol was mildly elevated at 6.1 mmol/L (normal range: 0-5.2 mmol/L).

Differential diagnosis

The patient presented with a transient neurological event, including facial droop and speech difficulties, with bilateral white matter changes, raising the possibility of several diagnoses, including ischemic stroke, given the presence of a restricted diffusion lesion in the right internal capsule, and periventricular white matter changes consistent with an ischemic event. Similarly, MRI demonstrated white matter hyperintensities and bilateral periventricular changes, and in the context of a stroke-like episode, this raised suspicion of CADASIL. Moreover, severely elevated blood pressure could also suggest hypertensive encephalopathy; however, the MRI findings were more consistent with ischemic changes rather than the edema typically associated with hypertensive crises.

Management

The patient was started on antihypertensive therapy, and analgesia was administered. He was also commenced on dual antiplatelet therapy with aspirin 75 mg and clopidogrel 75 mg. Blood pressure was optimised using ramipril 5 mg and amlodipine 10 mg, along with atorvastatin 80 mg. He was also referred to the stroke team to explore the possibility of CADASIL and other underlying vascular disorders. Based on the MRI findings, long-term follow-up with repeat imaging and genetic testing was recommended to confirm the diagnosis of CADASIL. Follow-up will be arranged to review the genetic test results. As of the most recent follow-up, the patient had shown clinical improvement, with resolution of his headache and no new neurological deficits.

## Discussion

Stroke due to underlying conditions such as CADASIL, particularly in adults, is rare [2]. However, our patient, in his early 40s, presented with neurological deficits and white matter changes on imaging, along with a clinical presentation consistent with transient ischemic events, which should raise suspicion for underlying conditions such as CADASIL. The typical presentation of CADASIL includes migraine with aura, the earliest feature of the disease, transient ischemic attack (TIA), recurrent ischemic strokes, and psychiatric disturbances, such as mood disorders including major depression, bipolar disorder, mania, dysthymia, and suicidal attempts, as well as cognitive impairment that may progress to dementia, along with Parkinsonian and other movement disorders. Patients may experience these symptoms even in the absence of hypertension, diabetes, or smoking [3,4]. However, our patient, with a history of diabetic retinopathy, presented with sudden-onset severe frontal and occipital headache, slurred speech, drooling of saliva, a sensation of a tied tongue, left-sided facial droop, right-sided weakness, and elevated blood pressure, but these symptoms gradually resolved.

This cerebrovascular encephalopathy on radiological investigation typically shows bilateral symmetrical white matter hyperintensities, especially in the anterior temporal lobes, frontal lobes, and periventricular areas, and less commonly in the external capsule and corpus callosum. Lacunar infarcts may also be observed in the centrum semiovale, basal ganglia, thalamus, and pons [5]. Genetic testing is considered the gold standard for diagnosis, but CADASIL can also be diagnosed clinically based on a detailed history and radiological findings [6]. Our patient’s CT head showed no acute intracranial hemorrhage or recent infarction, but bilateral periventricular white matter hypodensities were noted. Similarly, MRI of the brain demonstrated bilateral periventricular and deep white matter changes, with a small area of restricted diffusion adjacent to the posterior limb of the right internal capsule, consistent with a small ischemic infarct. These radiological findings further strengthened the likelihood of a CADASIL diagnosis. Blood tests also revealed elevated cholesterol levels.

To date, no treatment exists that can reverse or cure this illness. However, management of CADASIL primarily focuses on symptom relief and prevention of complications. Migraine with aura may be treated with acetaminophen or non-steroidal anti-inflammatory drugs (NSAIDs), and other prophylactic therapies may also be considered. Aspirin and other antiplatelet agents are used to reduce the risk of stroke. Supportive care and cognitive rehabilitation should be offered to patients with cognitive impairment or dementia. Similarly, antidepressants and mood stabilisers are recommended for mood and psychiatric symptoms. As CADASIL is an autosomal dominant condition, genetic counseling is an important component of management [7,8]. However, our patient was managed with antihypertensive therapy and analgesia, and was later commenced on dual antiplatelet therapy with aspirin 75 mg and clopidogrel 75 mg. Blood pressure was optimised using ramipril 5 mg and amlodipine 10 mg, along with atorvastatin 80 mg. He was also referred for a stroke consultation to explore the possibility of CADASIL and other underlying vascular conditions.

Given the MRI findings, long-term follow-up with repeat imaging and genetic testing was recommended to confirm or exclude CADASIL. The stroke team will review the genetic test results. The patient underwent an interval MRI at four months, and persistent white matter changes were noted. As of the most recent follow-up, the patient had shown clinical improvement, with resolution of his headache and no new neurological deficits. This follow-up highlights that early recognition and appropriate management are crucial to prevent further neurological damage, and timely referral to neurology is essential for the optimal management of these patients.

## Conclusions

This report emphasises the importance of early imaging in young patients presenting with neurological deficits, even when symptoms resolve spontaneously. White matter changes on MRI, combined with the patient’s transient neurological symptoms, strongly suggest an underlying cause such as CADASIL. Therefore, prompt identification and management are crucial to prevent further neurological damage, and referral to a stroke service is essential for appropriate care. Additionally, further investigations, including genetic testing and long-term follow-up, are recommended to confirm the diagnosis.
